# Identification of ERBB Pathway-Activated Cells in Triple-Negative Breast Cancer

**DOI:** 10.5808/GI.2019.17.1.e3

**Published:** 2019-03-31

**Authors:** Soo Young Cho

**Affiliations:** Clinical Genomics Analysis Branch, National Cancer Center, Goyang 10408, Korea

**Keywords:** breast neoplasms, ERBB signaling pathway, intratumor heterogeneity, molecular subtyping, single-cell RNA-seq

## Abstract

Intratumor heterogeneity within a single tumor mass is one of the hallmarks of malignancy and has been reported in various tumor types. The molecular characterization of intratumor heterogeneity in breast cancer is a significant challenge for effective treatment. Using single-cell RNA sequencing (RNA-seq) data from a public resource, an ERBB pathway activated triple-negative cell population was identified. The differential expression of three subtyping marker genes (ERBB2, ESR1, and PGR) was not changed in the bulk RNA-seq data, but the single-cell transcriptomes showed intratumor heterogeneity. This result shows that ERBB signaling is activated using an indirect route and that the molecular subtype is changed on a single-cell level. Our data propose a different view on breast cancer subtypes, clarifying much confusion in this field and contributing to precision medicine.

## Introduction

Breast cancer is the most common cancer in women, and it is estimated that over 508,000 women have died due to breast cancer [1]. Five main intrinsic or molecular subtypes of breast cancer have been proposed: luminal A, luminal B, triple-negative (TNBC)/basal-like, human epidermal growth factor receptor 2 (HER2)-enriched (HER2+), and normal-like [2, 3]. These breast cancer molecular subtypes have different gene expression patterns, clinical features, treatments, and prognoses.

Chung et al. [4] proposed a single-cell-level breast cancer transcriptome and identified a clinically important subpopulation. To understand intratumor heterogeneity expression patterns in breast cancer, a breast cancer cell population from single-cell RNA-seq (scRNA) data was analyzed from a public database.

The HER2+-like TNBC subtype from GSE75688 was identified. ERBB2 expression in a BC07 triple-negative patient was not significantly different in bulk-RNA and scRNA data. PIK3CB and RAF1 were highly expressed in the cell population in the BC07 patient. Our data propose a different view on breast cancer subtypes, clarifying much confusion in this field and contributing to precision medicine.

## Methods

### Data analysis

scRNA data for breast cancer were downloaded from GSE75688, which contains 549 primary breast cancer cells for 11 patients with estrogen receptor (ER)-positive (BC01, BC02), ER- and HER2-positive (BC03), lymph node metastasis (BC03LN), HER2-positive (BC04, BC05, BC06), triple-negative breast cancer (BC07-BC11), and matched bulk tumors [4]. Data for 11 primary breast cancers, excluding the metastasis sample, were selected (Supplementary Table 1). The reads were aligned to the human reference genome hg19 using STAR 2-pass mode and fragments per kilobase million (FPKM), calculated by RSEM. The cells contained more than 70% unique mapping reads. Genes with an average FPKM of more than 2.5 across all cells were selected, and the average FPKM was more than 4 in the same sample. Finally, in total, 415 cells and 4,269 genes were selected.

### Statistical analyses

R package was used for all statistical analyses. Data were analyzed using student’s ttest. A p-value < 0.05 was considered statistically significant.

## Results

### Tumor cell clustering in breast cancer

**Fig. 1.**
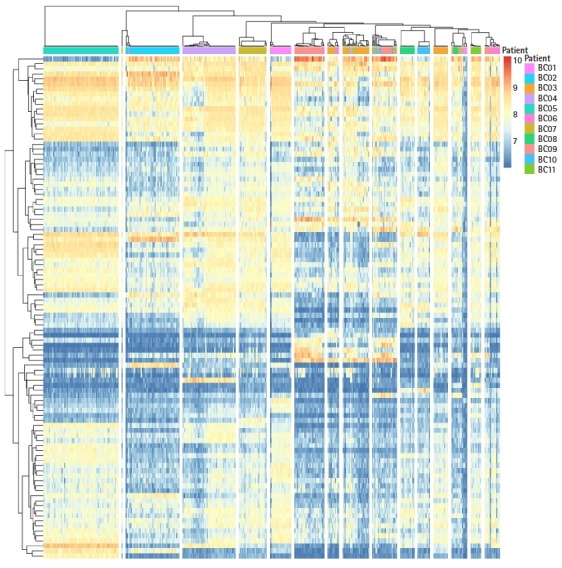
Hierarchical clustering for 515 single cells and 4,269 genes.

To understand subtype-specific expression patterns, hierarchical clustering method was used for 4,269 genes and 415 cells. All cells were clustered into 16 groups (Fig. 1). However, BC07 patient tumor cells were divided in two groups in the clustering result. To identify functional differences in BC07 patient tumor cells, Gene Set Enrichment Analysis was performed, and the ERBB signaling pathway was enriched in a subpopulation of BC07 patient tumor cells. ERBB signaling was predominantly manifested by the ERBB2 gene in the HER2+ subtype, not in TNBC. The BC07 patient had a heterogeneous intratumor expression pattern regarding the HER2+ and TNBC type.

### ERBB signaling pathway activation cells in TNBC

**Fig. 2.**
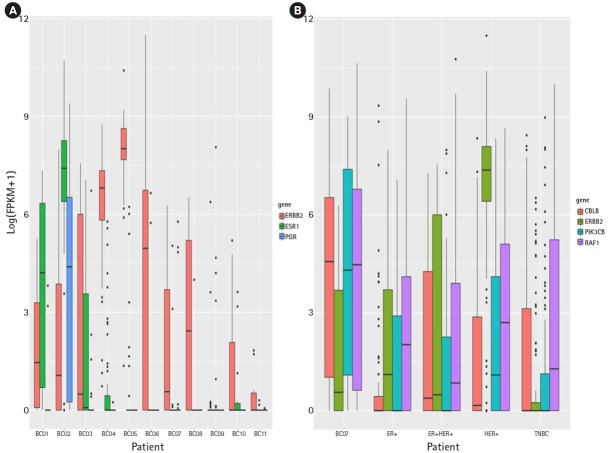
Breast cancer marker gene expression in bulk RNA sequencing (RNA-seq) and single-cell RNA-seq. (A) Breast cancer marker gene expression pattern in bulk RNA-seq (red, ERBB2; green, ESR1; and blue, PGR). (B) ERBB pathway activation marker gene expression pattern in selected cells (red, CBLB; green, ERBB2; blue, PIK3CB; and purple, RAF1). ER, estrogen receptor; HER2, human epidermal growth factor receptor 2; TNBC, triple-negative breast cancer.

To validate HER2+ pathway activated TNBC for the breast cancer subtype, marker gene expression was evaluated with bulk RNA-seq and single cell RNA-seq data. Three subtyping marker genes (ERBB2, ESR1, and PGR) were selected [5]. In the bulk RNA-seq data, BC07 showed low expression for ERBB2, ESR1, and PGR (Fig. 2A). In the scRNA data, ERBB2 expression in HER2+ likely cells from BC07 was lower than in other HER2+ subtype patients (2.2e16) and not higher than the other TNBC patients (p> 0.01) (Fig. 2B). The subtyping marker gene expression pattern in the bulk RNA-seq and scRNA data looked like that of the TNBC subtype. However, ERBB pathway regulators (PIK3CB and RAF1) were highly expressed in HER2+ likely cells. PIK3CB and RAF1 have been reported to be ERBB signaling pathway regulators and activators in cancer [6, 7]. The expression of PIK3CN and RAF1 was increased in HER2+ likely cells and activated (p<0.01). This result shows that the ERBB signaling pathway was activated in the TNBC subtype but that the mechanism was different from the HER+ subtype.

## Discussion

In conclusion, this result shows that ERBB signaling in TNBC is activated using an indirect route. Finally, the ERBB signaling pathway was activated in TNBC. Our data propose a different view on breast cancer subtypes, which clarifies much confusion in this field and contributes to precision medicine. Hence, further insights into the biology of ERBB activation TNBC should be forthcoming through additional studies.

## Supplementary Materials

Supplementary Table 1. Summary of selected samples
