# Supplementary material for: Identification of ERBB Pathway-Activated Cells in Triple-Negative Breast Cancer
Source: Genomics Inform. 2019 Mar 31;17(1):e3. doi: 10.5808/GI.2019.17.1.e3 (PMC6459170; doi:10.5808/GI.2019.17.1.e3)
Supplement: Supplementary Table 1. — Summary of selected samples [file gi-2019-17-1-e3-suppl1.pdf]

 GENOMICS & INFORMATICS

Supplementary Table 1. Summary of selected samples

GEO ID	Patient ID	Molecular type
GSE75688	BC01	ER-positive
	BC02	ER-positive
	BC03	ER- and HER2-positive
	BC04	HER2-positive
	BC05	HER2-positive
	BC06	HER2-positive
	BC07	Triple-negative
	BC08	Triple-negative
	BC09	Triple-negative
	BC10	Triple-negative
	BC11	Triple-negative

ER, estrogen receptor; HER2, human epidermal growth factor receptor 2.

https://doi.org/10.5808/GI.2019.17.1.e3
